# Supplementary material for: CLN3 deficiency leads to neurological and metabolic perturbations during early development
Source: Life Sci Alliance. 2024 Jan 9;7(3):e202302057. doi: 10.26508/lsa.202302057 (PMC10776888; doi:10.26508/lsa.202302057)
Supplement: Supplementary file 1 [file LSA-2023-02057_Supplemental_Data_1.docx]

**Supplementary File 1:** **Sanger sequencing of PCR amplicons in zebrafish *cln3* morphants**

**Figure from panel Fig. 1D**


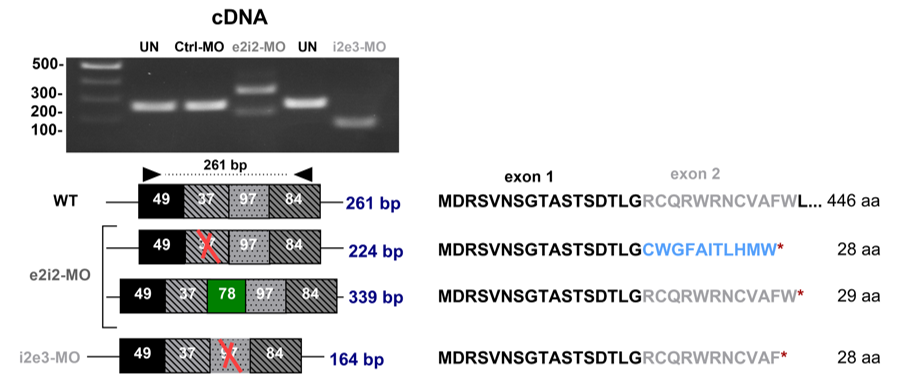


Validation of MO efficiency by PCR amplification of target regions in cDNA of the *cln3* morphants in comparison to uninjected (UN) and control morpholino (Ctrl-MO) injected larvae.

**cDNA sequencing results**

**e2i2-MO heavy band:**
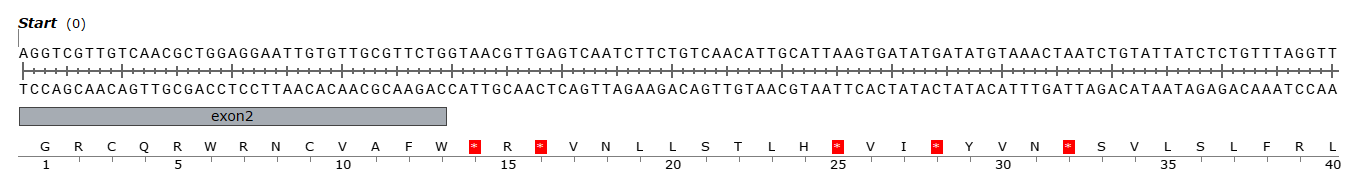
**e2i2-MO light band:**


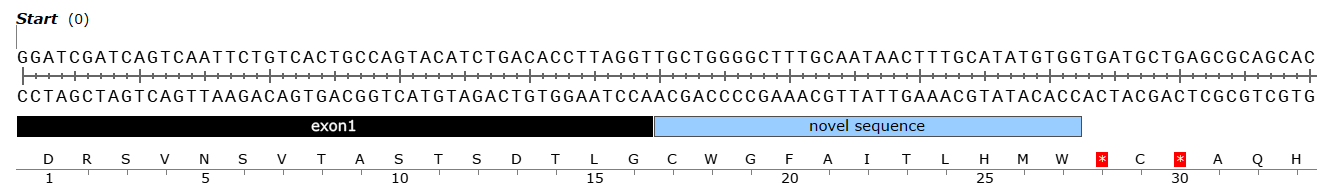


**i2e3-MO band:**
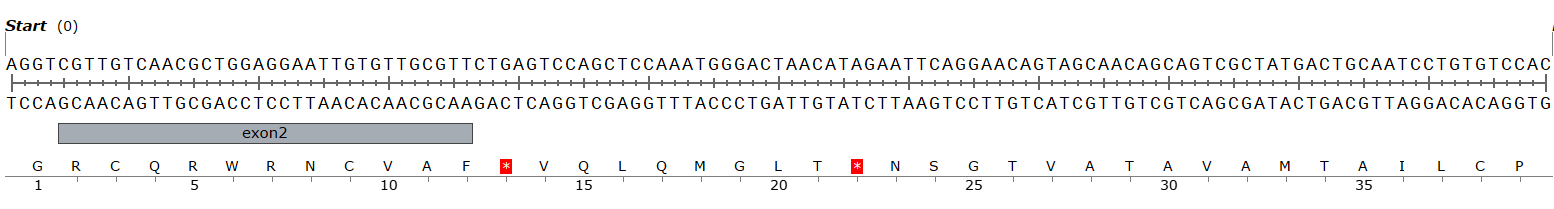


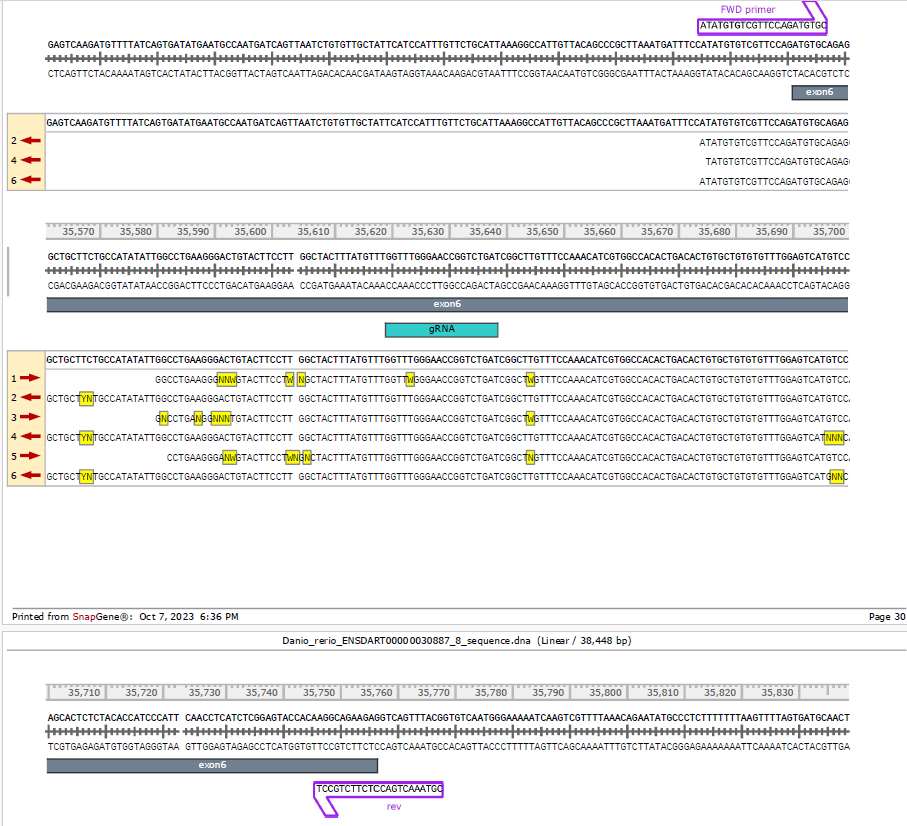


Multiple DNA alignment of PCR amplicons against the *slc45a2* reference sequence (ENSDART00000030887_8). 1, MUT1-Fwd; 2, MUT1-rev; 3, MUT2-Fwd; 4, MUT2-rev; 5, WT(AB)-fwd; 6, WT(AB)-rev.
