# Supplementary material for: CLN3 deficiency leads to neurological and metabolic perturbations during early development
Source: Life Sci Alliance. 2024 Jan 9;7(3):e202302057. doi: 10.26508/lsa.202302057 (PMC10776888; doi:10.26508/lsa.202302057)
Supplement: Supplementary file 2 [file LSA-2023-02057_Supplemental_Data_2.docx]

**File S2:** **Sanger sequencing of transcripts detected in zebrafish CRISPR mutants**

Based on the canonical *cln3* transcript (ENSDART00000055170.4) described in Ensembl, a primer pair was designed to amplify the entire coding sequence following the Gateway guidelines. Total RNA was extracted from 5 dpf WT, MUT1, and MUT2 larvae and cDNA was synthesized. The PCR reaction was analyzed by agarose gel electrophoresis (see below). Amplicons were cloned into pDONR221 and recombinant plasmid was sequenced using M13 primers (see below). Predicted transcript length: WT, 1342 bp; MUT1, 1440 bp; MUT2, 1259 bp.

**
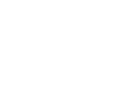

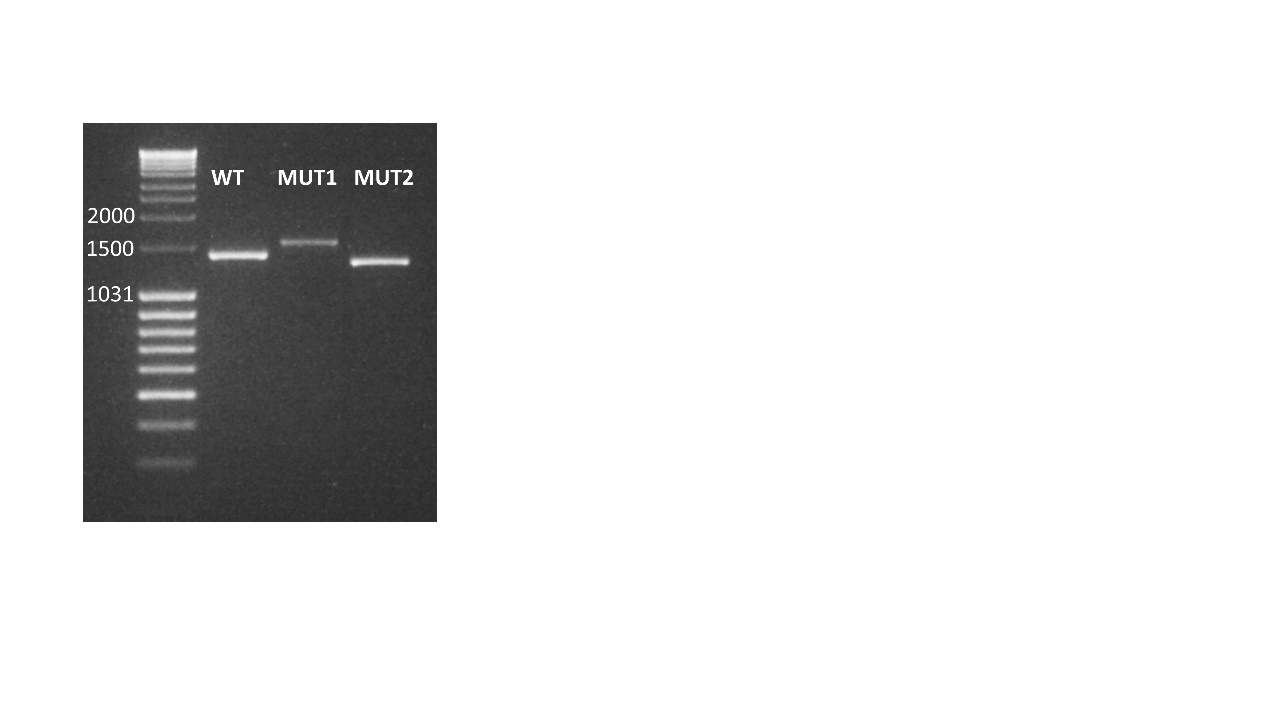
**

Agarose gel (2%) showing cln3 amplicons amplified from WT, MUT1 and MUT2 cDNA.

WT cDNA (1342 bp)

ATGGATCGATCAGTCAATTCTGTCACTGCCAGTACATCTGACACCTTAGGTCGTTGTCAACGCTGGAGGAATTGTGTTGCGTTCTGGTTGCTGGGGCTTTGCAATAACTTTGCATATGTGGTGATGCTGAGCGCAGCACATGACATCTTACAGAAACAAGAGTCTCAAAACACAACAGCACCTAGTCCAGCTCCAAATGGGACTAACATAGAATTCAGGAACAGTAGCAACAGCAGTCGCTATGACTGCAATCCTGTGTCCACTGCTGCTGTGCTGTTGGCTGATATCCTACCCACCCTATTAATCAAACTCACAACGCCTTTCTACATACACAAAGTGCCTTATGGATTCCGTGTTTTGGTGTGTTTCTTTACGGCAGTAGTCAGCTTCCTGATGGTGTCATTTTCATCAACAATATCGATGAGCATAATTGGTGTCATCTTTGCAAGTGTTAGTTCAGGATTGGGAGAACTTTCCTTCCTCTCGCTCTCAGTGTTTTTCAGCAGTGACGTGTTAAGTGGCTGGGGGTCCGGCACAGGGGCAGCAGGAGTCGCTGGAGCTTTACTCTACTCTGCATTAACACAGGCTGGCCTGACCCCACAAGTTACTCTATGGATCATGCTGGTGGTCCCTGTTATTTTGGCTGTTAGCTATTTTGTCCTCCTGGTGTTTCCGCACTCATTTCCACAGTGGAGATGCCCCGAGGTTAGTCAGAGCCTTTCCAGAGGGCTGAACTCTGAGGAGAGACGAGCTTTAATTGAGGAGGACACGGATACAGATGAGGACTCTGAACCAGCTCTGGAAGATCAAGATGACAAACACATCGGACCCCTGACCTTCACTGAGAAAAAATACATCATTAAGGGTCTGTTGAAGTTTATTTTTCCTCTTGCTTTGGTTTACTTTGCTGAGTATTTCATCAACCAAGGACTGATGGAGCTACTCTACTTTCCTGACTCCAGACTTTCACACGCTGAACAATATCGCTGGTATCAGACAGTTTATCAGATTGGAGTGTTTGTGTCGCGTACGTCTCTCTTCTGTTTTAAAATCAGGAAGATTGTTCTCATGTCCCTCTTACAGTGTGCCAATGCTGTTCTGTTGGTATTTGCCGTGTATTACCAGTTCTTGCCAAACATATCTGTGGTGTTTGTTATTATTGCCTTTGAGGGTCTATTGGGAGGAGCAGCATATGTAAATACCTTCTTTTTCATCAGGGAGGAGAGTGTTGAGCGGGAGAGAGAGTTTGCGATGGCCACAGCCACCGTAGGGGACAGTCTGGGAATTGCTTTTTCTGCAGCTGCTGCCTTCCCCGTTCACCATTATTTCTGCTCTTTATGAG

WT protein (446aa)

MDRSVNSVTASTSDTLGRCQRWRNCVAFWLLGLCNNFAYVVMLSAAHDILQKQESQNTTAPSPAPNGTNIEFRNSSNSSRYDCNPVSTAAVLLADILPTLLIKLTTPFYIHKVPYGFRVLVCFFTAVVSFLMVSFSSTISMSIIGVIFASVSSGLGELSFLSLSVFFSSDVLSGWGSGTGAAGVAGALLYSALTQAGLTPQVTLWIMLVVPVILAVSYFVLLVFPHSFPQWRCPEVSQSLSRGLNSEERRALIEEDTDTDEDSEPALEDQDDKHIGPLTFTEKKYIIKGLLKFIFPLALVYFAEYFINQGLMELLYFPDSRLSHAEQYRWYQTVYQIGVFVSRTSLFCFKIRKIVLMSLLQCANAVLLVFAVYYQFLPNISVVFVIIAFEGLLGGAAYVNTFFFIREESVEREREFAMATATVGDSLGIAFSAAAAFPVHHYFCSL*

MUT1 cDNA (1447 bp)

ATGGATCGATCAGTCAATTCTGTCACTGCCAGTACATCTGACACCTTAGGTCGTTGTCAACGCTGGAGGAATTGTGTTGCGTTCTGGTTGCTGGGGCTTTGCAATAACTTTGCATATGTGGTGATGCTGAGCGCAGCACATGACATCTTACAGAAACAAGAGTCTCAAAACACAACAGCACCTAGTCCAGCTCCAAATGGGACTAACATAGAATTCAGGAACAGTAGCAACAGCAGTCGCTATGACTGCAATCCTGTGTCCACTGTTGTTTTCTGCTGTGCTGTCTCTCTTTCTAGGCTGTGCTGTCTTTCTAGGCTGTGCTGTTGGCTTCTGCTGTGCTGTTGGCTGATATCCTACCCACCCTATTAATCAAACTCACAGCGCCTTTCTACATACACAAAGTGCCTTATGGGCTGTGCTGTTGGCTGATATCCTACCCACCCTATTAATCAAACTCACAGCGCCTTTCTACATACACAAAGTGCCTTATGGATTCCGTGTTTTGGTGTGTTTCTTTACGGCAGTAGTCAGCTTCCTGATGGTGTCATTTTCATCAACAATATCGATGAGCATAATTGGTGTCATCTTTGCAAGTGTTAGTTCAGGATTGGGAGAACTTTCCTTCCTCTCGCTCTCAGTGTTTTTCAGCAGTGACGTGTTAAGTGGCTGGGGGTCCGGCACAGGGGCAGCAGGAGTCGCTGGAGCTTTACTCTACTCTGCATTAACACAGGCTGGCCTGACCCCACAAGTTACTCTATGGATCATGCTGGTGGTCCCTGTTATTTTGGCTGTTAGTGGAGATGCCCCGAGGTTAGTCAGAGCCTTTCCAGAGGGCTGAACTCTGAGGAGAGACGAGCTTTAATTGAGGAGGACACGGATACAGATGAGGACTCTGAACCAGCTCTGGAAGATCAAGATGACAAACACATCGGACCCCTGACCTTCACTGAGAAAAAATACATCATTAAGGGTCTGTTGAAGTTTATTTTTCCTCTTGCTTTGGTTTACTTTGCTGAGTATTTCATCAACCAAGGACTGATGGAGCTACTCTACTTTCCTGACTCCAGACTTTCACACGCTGAACAATATCGCTGGTATCAGACAGTTTATCAGATTGGAGTGTTTGTGTCGCGTACGTCTCTCTTCTGTTTTAAAATCAGGAAGATTTTTCTCATGTCCCTCTTACAGTGTGCCAATGCTGTTCTGTTGGTATTCGCGGTGTATTACCAGTTCTTGCCAAACATATCTGTGGTGTTTGTTATTATTGCCTTTGAGGGTCTATTGGGAGGAGCAGCATATGTAAATACCTTCTTTTTCATCAGGGAGGAGAGTGTTGAGCGGGAGAGAGAATTTGCGATGGCCACAGCCACCGTAGGGGACAGTCTGGGAATTGCTTTTTCTGCAGCTGCTGCCTTCCCCGTTCACCATTATTTCTGCTCTTTATGAG

MUT1 protein (122 aa)

MDRSVNSVTASTSDTLGRCQRWRNCVAFWLLGLCNNFAYVVMLSAAHDILQKQESQNTTAPSPAPNGTNIEFRNSSNSSRYDCNPVSTVVFCCAVSLSRLCCLSRLCCWLLLCCWLISYPPY*

MUT2 cDNA (1259 bp)

>ATGGATCGATCAGTCAATTCTGTCACTGCCAGTACATCTGACACCTTAGGTCGTTGTCAACGCTGGAGGAATTGTGTTGCGTTCTGGTTGCTGGGGCTTTGCAATAACTTTGCATATGTGGTGATGCTGAGCGCAGCACATGACATCTTACAGAAACAAGAGTCTCAAAACACAACAGCACCTGCTGTGCTGTTGGCTGATATCCTACCCACCCTATTAATCAAACTCACAGCGCCTTTCTACATACACAAAGTGCCTTATGGATTCCGTGTTTTGGTGTGTTTCTTTACGGCAGTAGTCAGCTTCCTGATGGTGTCATTTTCATCAACAATATCGATGAGCATAATTGGTGTCATCTTTGCAAGTGTTAGTTCAGGATTGGGAGAACTTTCCTTCCTCTCGCTCTCAGTGTTTTTCAGCAGTGACGTGTTAAGTGGCTGGGGGTCCGGCACAGGGGCAGCAGGAGTCGCTGGAGCTTTACTCTACTCTGCATTAACACAGGCTGGCCTGACCCCACAAGTTACTCTATGGATCATGCTGGTGGTCCCTGTTATTTTGGCTGTTAGCTATTTTGTCCTCCTGGTGTTTCCGCACTCATTTCCACAGTGGAGATGCCCCGAGGTTAGTCAGAGCCTTTCCAGAGGGCTGAACTCTGAGGAGAGACGAGCTTTAATTGAGGAGGACACGGATACAGATGAGGACTCTGAACCAGCTCTGGAAGATCAAGATGACAAACACATCGGACCCCTGACCTTCACTGAGAAAAAATACATCATTAAGGGTCTGTTGAAGTTTATTTTTCCTCTTGCTTTGGTTTACTTTGCTGAGTATTTCATCAACCAAGGACTGATGGAGCTACTCTACTTTCCTGACTCCAGACTTTCACACGCTGAACAATATCGCTGGTATCAGACAGTTTATCAGATTGGAGTGTTTGTGTCGCGTACGTCTCTCTTCTGTTTTAAAATCAGGAAGATTGTTCTCATGTCCCTCTTACAGTGTGCCAATGCTGTTCTGTTGGTATTTGCCGTGTATTACCAGTTCTTGCCAAACATATCTGTGGTGTTTGTTATTATTGCCTTTGAGGGTCTATTGGGAGGAGCAGCATATGTAAATACCTTCTTTTTCATCAGGGAGGAGAGTGTTGAGCGGGAGAGAGAGTTTGCGATGGCCACAGCCACCGTAGGGGACAGTCTGGGAATTGCTTTTTCTGCAGCTGCTGCCTTCCCCGTTCACCATTATTTCTGCTCTTTATGAG <

MUT2 protein (418 aa)

MDRSVNSVTASTSDTLGRCQRWRNCVAFWLLGLCNNFAYVVMLSAAHDILQKQESQNTTAPAVLLADILPTLLIKLTAPFYIHKVPYGFRVLVCFFTAVVSFLMVSFSSTISMSIIGVIFASVSSGLGELSFLSLSVFFSSDVLSGWGSGTGAAGVAGALLYSALTQAGLTPQVTLWIMLVVPVILAVSYFVLLVFPHSFPQWRCPEVSQSLSRGLNSEERRALIEEDTDTDEDSEPALEDQDDKHIGPLTFTEKKYIIKGLLKFIFPLALVYFAEYFINQGLMELLYFPDSRLSHAEQYRWYQTVYQIGVFVSRTSLFCFKIRKIVLMSLLQCANAVLLVFAVYYQFLPNISVVFVIIAFEGLLGGAAYVNTFFFIREESVEREREFAMATATVGDSLGIAFSAAAAFPVHHYFCSL*


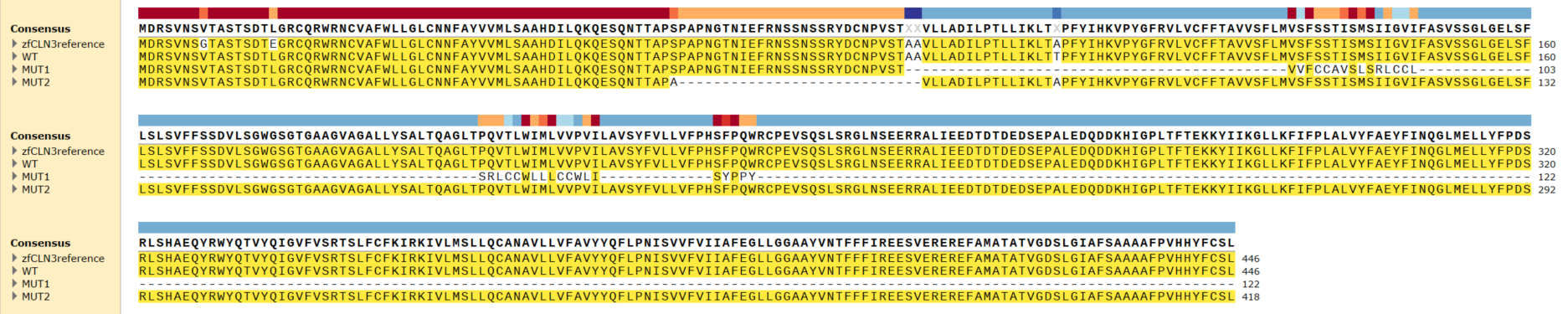


Multiple sequence alignment of the Cln3 reference sequence (ENSDART00000055170) with the Cln3 sequences of our WT AB reference strain, and of the MUT1 and MUT2 lines (predicted protein sequences based on the cDNA sequences) using theMUSCLE algorithm in the SnapGene software (V6.2.2). Strictly conserved residues are highlighted in yellow and the color-coded bar above the alignment indicates the conservation degree.
