# Supplementary material for: CLN3 deficiency leads to neurological and metabolic perturbations during early development
Source: Life Sci Alliance. 2024 Jan 9;7(3):e202302057. doi: 10.26508/lsa.202302057 (PMC10776888; doi:10.26508/lsa.202302057)
Supplement: Supplementary file 3 [file LSA-2023-02057_Supplemental_Data_3.docx]

**File S3:** **Sanger sequencing of the CRISPR target site in the *slc45a2* locus**

Genomic DNA of brains of F2 adult WT and MUT2 fish and of a pool of 40 MUT1 larvae (F3) at 5 dpf was extracted. PCR was performed with primers surrounding the CRISPR target sequence (*slca45a2*_fwd:TATGTGTCGTTCCAGATGTGC, *slc45a2*_rev: CGTAAACTGACCTCTTCTGCCT) and the reactions were analyzed by agarose gel electrophoresis (see below). In all samples, a single band was observed. The bands were cut out from the gel, purified, and amplicons were sent for sequencing using the forward and reverse primers. As shown in the alignment figure below, no mutations were observed consistently in both read directions showing that *cln3* mutant fish do not carry a mutation in the *slc45a2* gene.


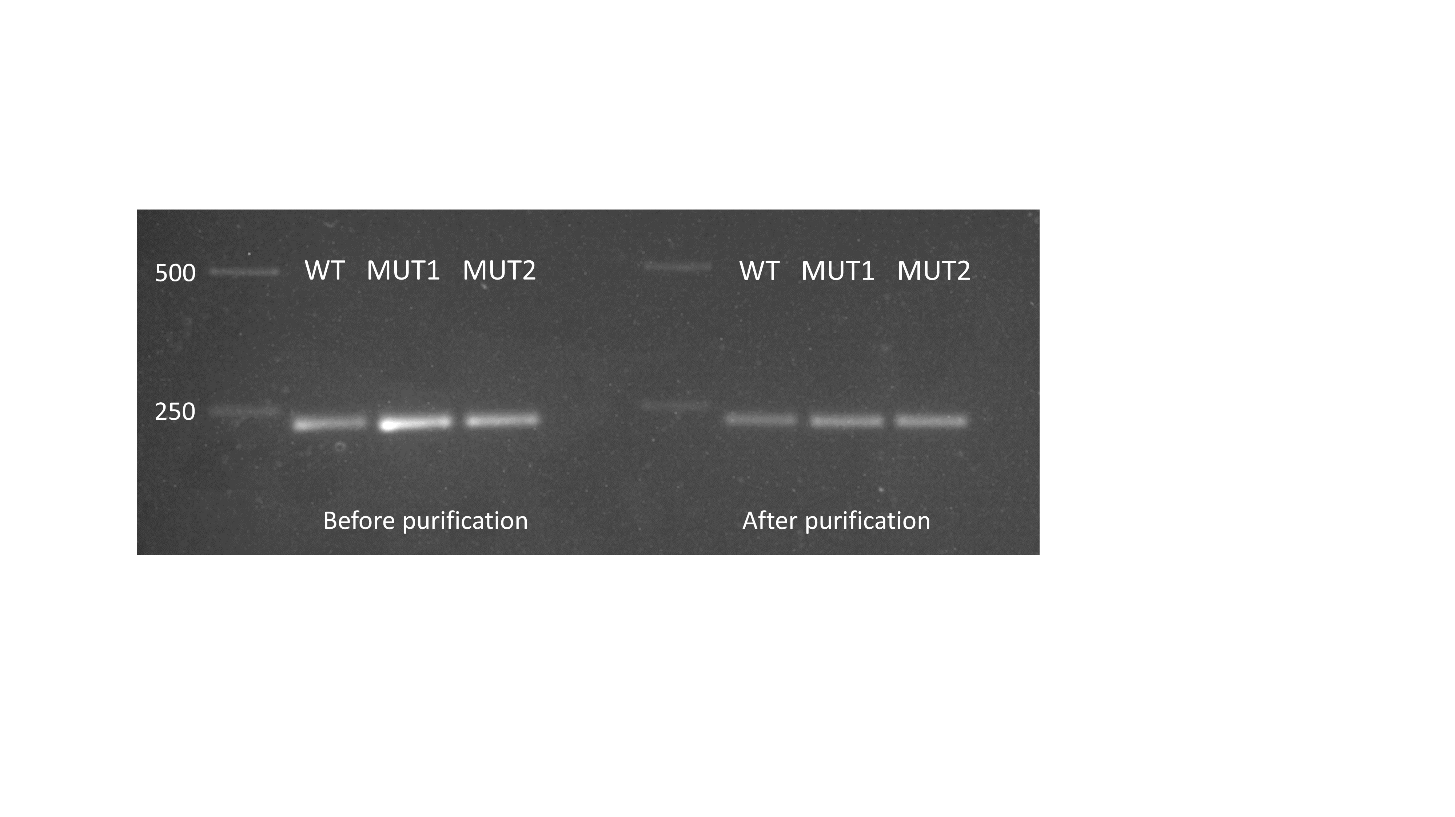


Agarose gel (3.5%) showing *slc45a2* amplicons (before and after gel purification) obtained by PCR on genomic DNA from WT (AB), MUT1, and MUT2 zebrafish.


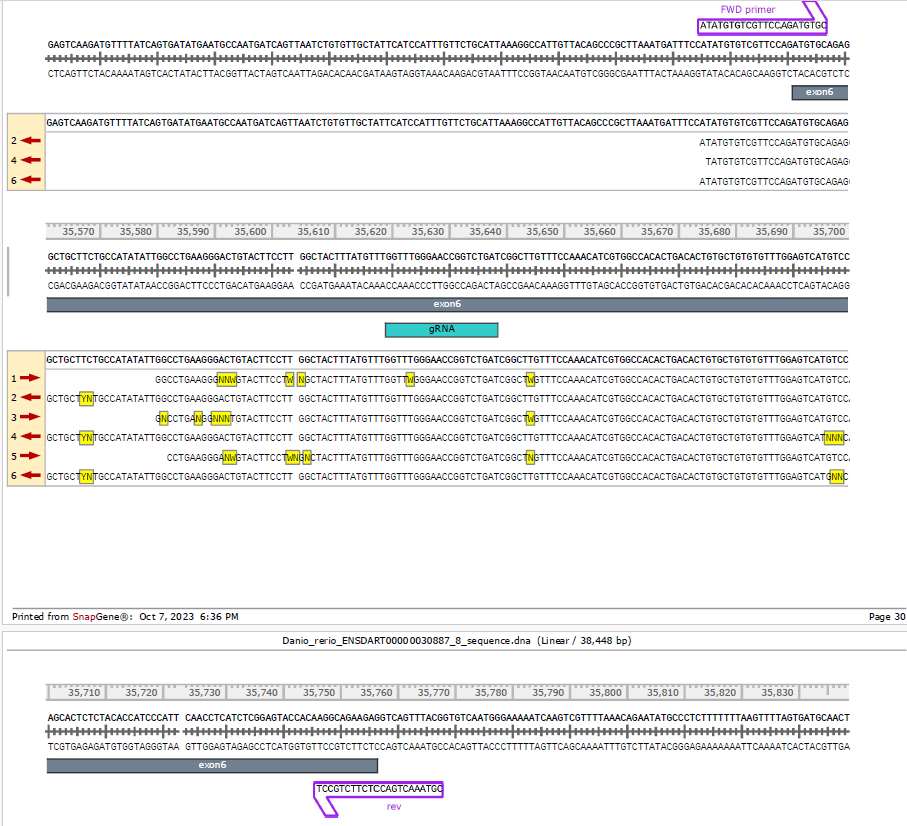
Multiple DNA alignment of PCR amplicons against the *slc45a2* reference sequence (ENSDART00000030887_8). 1, MUT1-Fwd; 2, MUT1-rev; 3, MUT2-Fwd; 4, MUT2-rev; 5, WT(AB)-fwd; 6, WT(AB)-rev.
