# Supplementary material for: CLN3 deficiency leads to neurological and metabolic perturbations during early development
Source: Life Sci Alliance. 2024 Jan 9;7(3):e202302057. doi: 10.26508/lsa.202302057 (PMC10776888; doi:10.26508/lsa.202302057)
Supplement: Supplementary file 8 [file LSA-2023-02057_TableS5.docx]

**S5 Table. Abbreviations used for statistically significant differential lipid subclasses in *cln3* knockout *versus* wild-type larvae**

| **Lipid subclass** | **Abbreviation** |
| --- | --- |
| Carnitine | CAR |
| Diacylglycerol | DG |
| Triacylglycerol | TG |
| Lysophophatidylcholine | LPC |
| Phosphatidylcholine | PC |
| Phosphatidylglycerol | PG |
| Vitamin A fatty acid ester | VAE |
| Hexosylceramide | HexCer |
| Sphingomyelin | SM |
| Bismonoacylglycerophosphate | BMP |
| Ceramide phosphoethanolamine | Pe-Cer |
| Free fatty acid | FA |
| Acyl steryl glycoside | ASG |
