# Supplementary material for: CLN3 deficiency leads to neurological and metabolic perturbations during early development
Source: Life Sci Alliance. 2024 Jan 9;7(3):e202302057. doi: 10.26508/lsa.202302057 (PMC10776888; doi:10.26508/lsa.202302057)
Supplement: Supplementary file 9 [file LSA-2023-02057_Supplemental_Data_4.docx]

| **Line name** | **Generation** | **Experiment** | **Figure** |
| --- | --- | --- | --- |
| MUT1 | F3 | Behavioral assay | 3, S4 |
| MUT2 | F3 | Behavioral assay | 3, S5, S8 |
| MUT1 | F5 | Untargeted metabolomics Experiment 1 | 4, 5, S5 |
| MUT1 | F5 | Untargeted metabolomics Experiment 2 | S6, S7, S8 |
| MUT1 | F9 | Targeted LC-MS Experiment 1 | 6A, S10,S11 |
| MUT1 | F9 | Targeted LC-MS Experiment 2 | 6A,D |
| MUT2 | F12 | Targeted LC-MS Experiment 2 | 6A,D |

**File S4. List of F-generation used in each experiment.**
