# Supplementary material for: CLN3 deficiency leads to neurological and metabolic perturbations during early development
Source: Life Sci Alliance. 2024 Jan 9;7(3):e202302057. doi: 10.26508/lsa.202302057 (PMC10776888; doi:10.26508/lsa.202302057)
Supplement: Supplementary file 10 [file LSA-2023-02057_TableS6.docx]

**S6 Table. List of primers used in this study**

| **Primer name** | **Sequence (5’ to 3’ direction)** | **Purpose** |
| --- | --- | --- |
| CLN3_exon1.fwd | GGGATCGATCAGTCAATTCTG | SB–MO validation |
| CLN3_exon4.rev | GGTGGACACAGGATTGCAGTC | SB-MO validation |
| Zfcln3_int.rev | ATTGACACTGAAATCAATGGCA | *cln*3 knockout genotyping |
| OZFF-007 | AAAAGCACCGACTCGGTGCCACTTTTTCAAGTTGATAACGGACTAGCCTTATTTTAACTTGCTATTTCTAGCTCTAAAAC | Constant oligo CRISPR |
| OZFF-008 | GAAGACAGACTTACAGCAGGTTTTAGAGCTAGAAATAGCAAG | cln3 gene-specific oligo for sgRNA |
| OZFF-009 | TCGGTTCACTCACTAGAGTCCA | CRISPR efficiency testing |
| OZFF-010 | ATACGACAAGGCAACATGACAG | CRISPR efficiency testing |
| OZFF-020 | TAATACGACTCACTATAGGTTTGGGAACCGGTCTGATGTTTTAGAGCTAGAAATAGCAAG | Slc45a2 gene-specific oligo for sgRNA |
| Ef1α_fwd | CTGGAGGCCAGCTCAAACAT | HK gene for qPCR |
| Ef1α_rev | ATCAAGAAGAGTAGTACCGCTAGCATTAC | HK gene for qPCR |
| Rpl13α_fwd | TCTGGAGGACTGTAAGAGGTATGC | HK gene for qPCR |
| Rpl13α_rev | AGACGCACAATCTTGAGAGCAG | HK gene for qPCR |
| Cln3primer1_fwd | CCGAGGTTAGTCAGAGCCTT | qPCR |
| Cln3primer1_rev | AAGGTTAGGGGTCCGATGTG | qPCR |
| Cln3primer2_fwd | CACATCGGACCCCTAACCTT | qPCR |
| Cln3primer2_rev | GCGTGTGAAAGTCTGGAGTC | qPCR |
| cln3-exon2_fwd | GTCAACGCTGGAGGAATTGT | pGEMT-cloning |
| cln3-exon7_rev | CACTTGCAAAGATGACACCAA | pGEMT-cloning |
| Zfcln3N-fus_fwd | GGGGACAAGTTTGTACAAAAAAGCAGGCTTCATGGATCGATCAGTCAATTCTG | Gateway cloning |
| Zfcln3_NE_rev | GGGGACCACTTTGTACAAGAAAGCTGGGTCTCATAAAGAGCAGAAATAAT | Gateway cloning |
| slc45a2grna_fwd | ATATGTGTCGTTCCAGATGTGC | Slc45a2 Crispr target site |
| slc45a2grna_rev | CGTAAACTGACCTCTTCTGCCT | Slc45a2 Crispr target site |

*HK, Housekeeping gene
